# 1,4-Dimethyl­piperazine-1,4-diium bis­(hexa­fluoro­phosphate) dihydrate

**DOI:** 10.1107/S1600536812017229

**Published:** 2012-04-25

**Authors:** Su-Wen Sun

**Affiliations:** a College of Chemistry and Chemical Engineering, Southeast University, Nanjing 211189, People’s Republic of China

## Abstract

In the title hydrated mol­ecular salt, C_6_H_16_N_2_^+^·2PF_6_^−^·2H_2_O, the complete 1,4-dimethyl­piperazine-1,4-diium dication is generated by crystallographic inversion symmetry and both C—N bonds are in equatorial orientations. In the crystal, the components are linked by O—H⋯F and N—H⋯O hydrogen bonds but there are no direct links between cations and anions.

## Related literature

For background to mol­ecular ferroelectrics, see: Fu *et al.* (2009 ▶); Ye *et al.* (2006 ▶).
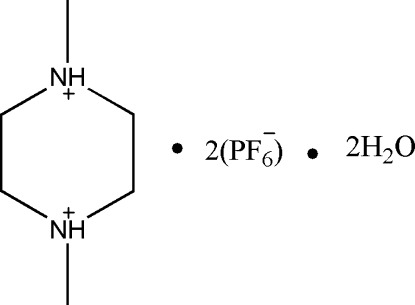


## Experimental

### 

#### Crystal data

C_6_H_16_N_2_^+^·2PF_6_^−^·2H_2_O
*M* *_r_* = 442.18
Monoclinic, 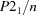

*a* = 8.1382 (16) Å
*b* = 6.3666 (13) Å
*c* = 15.758 (3) Å
β = 99.55 (3)°
*V* = 805.1 (3) Å^3^
*Z* = 2
Mo *K*α radiation
μ = 0.40 mm^−1^
*T* = 293 K
0.30 × 0.30 × 0.20 mm

#### Data collection

Rigaku Mercury CCD diffractometer
Absorption correction: multi-scan (*CrystalClear*; Rigaku, 2005 ▶) *T*_min_ = 0.489, *T*_max_ = 1.000
8084 measured reflections
1849 independent reflections
1093 reflections with *I* > 2σ(*I*)
*R*_int_ = 0.079

#### Refinement

*R*[*F* ^2^ > 2σ(*F* ^2^)] = 0.068
*wR*(*F* ^2^) = 0.163
*S* = 1.06
1849 reflections
122 parameters
3 restraints
H atoms treated by a mixture of independent and constrained refinement
Δρ_max_ = 0.37 e Å^−3^
Δρ_min_ = −0.36 e Å^−3^

### 

Data collection: *CrystalClear* (Rigaku, 2005 ▶); cell refinement: *CrystalClear*; data reduction: *CrystalClear*; program(s) used to solve structure: *SHELXS97* (Sheldrick, 2008 ▶); program(s) used to refine structure: *SHELXL97* (Sheldrick, 2008 ▶); molecular graphics: *SHELXTL* (Sheldrick, 2008 ▶); software used to prepare material for publication: *SHELXL97*.

## Supplementary Material

Crystal structure: contains datablock(s) I, global. DOI: 10.1107/S1600536812017229/hb6717sup1.cif

Structure factors: contains datablock(s) I. DOI: 10.1107/S1600536812017229/hb6717Isup2.hkl

Supplementary material file. DOI: 10.1107/S1600536812017229/hb6717Isup3.cml

Additional supplementary materials:  crystallographic information; 3D view; checkCIF report

## Figures and Tables

**Table 1:** Hydrogen-bond geometry (Å, °)

*D*—H⋯*A*	*D*—H	H⋯*A*	*D*⋯*A*	*D*—H⋯*A*
O1—H1⋯F6^i^	0.85 (1)	2.18 (2)	3.008 (5)	167 (5)
O1—H2⋯F3^ii^	0.84 (1)	2.42 (4)	2.923 (5)	119 (4)
N1—H3⋯O1^iii^	0.88 (4)	1.98 (4)	2.814 (4)	159 (4)

Symmetry codes: (i) 
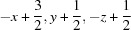
; (ii) 
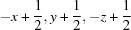
; (iii) 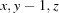.

**Fig. 1.**
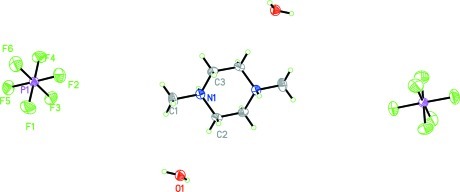
T The molecular structure of the title compound, showing the atomic numbering scheme with 30% probability displacement ellipsoids.

**Fig. 2.**
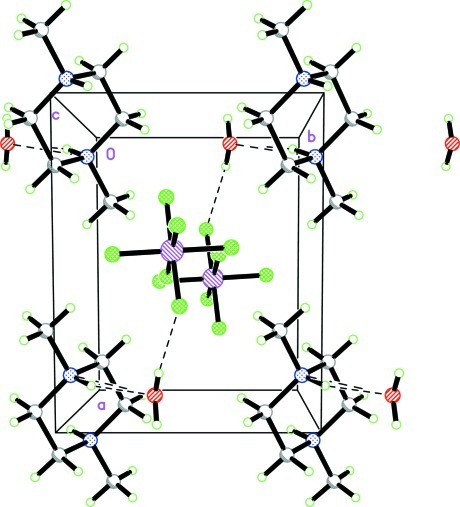
A view of the packing of the title compound, stacking along the b axis. Dashed lines indicate hydrogen bonds.

## supplementary crystallographic information

### Comment

Dielectric constant measurements of compounds as a function of temperature is the basic methods to find the materials which possess potential ferroelectric phase changes (Fu *et al.*,2009;Ye *et al.*,2006).The dielectric constant of the title compound has been measured, but showed no dielectric disuniformity in the range 120–385 K (mp. 393–402 K)

The asymmetric unit of the title compound is shown in Fig. 1. crystallized in the monoclinic P2(1)/n space group, The crystal packing Fig. 2 features O—H···F and N—H···O hydrogen bonds(O1—H1··· F6, O1—H2···F3, N1—H3···O1) between the C_6_H_16_N_2_^+^ cations and PF_6_^-^ anions and H_2_O (see; Table 1).

### Experimental

1,4-Dimethyl-piperazine (0.57 g) and an excess of hexafluorophosphoric acid (0.95 g) were dissolved in methanol without any precipitation under stirring at the ambient temperature. Colourless blocks of the title compound were obtained by slow evaporation at room temperature over two days.

### Refinement

H atoms were placed in calculated positions (N—H = 0.89 Å; C—H = 0.96 Å and 0.97 Å for C*sp^3^* atoms), assigned fixed *U*_iso_ values [1.5*U*eq(C*sp^3^*,*N*)] and allowed to ride.The H1 and H2 on the O1 were restrained with O—H =0.85 Å yielding O1—H1 = 0.8448 Å and O1 —H2 = 0.8440 Å, with *U*_iso_(H) = 1.2 *U*_iso_(O)

### Crystal data

**Table:**

C_6_H_16_N_2_^+^·2PF_6_^−^·2H_2_O	*Z* = 2
*M**_r_* = 442.18	*F*(000) = 448
Monoclinic, *P*2_1_/*n*	*D*_x_ = 1.824 Mg m^−^^3^
Hall symbol: -P 2yn	Mo *K*α radiation, λ = 0.71073 Å
*a* = 8.1382 (16) Å	θ = 3.0–27.5°
*b* = 6.3666 (13) Å	µ = 0.40 mm^−^^1^
*c* = 15.758 (3) Å	*T* = 293 K
β = 99.55 (3)°	Block, colorless
*V* = 805.1 (3) Å^3^	0.30 × 0.30 × 0.20 mm

### Data collection

**Table:**

Rigaku Mercury CCD diffractometer	1849 independent reflections
Radiation source: fine-focus sealed tube	1093 reflections with *I* > 2σ(*I*)
Graphite monochromator	*R*_int_ = 0.079
ω scans	θ_max_ = 27.5°, θ_min_ = 3.0°
Absorption correction: multi-scan (*CrystalClear*; Rigaku, 2005)	*h* = −10→10
*T*_min_ = 0.489, *T*_max_ = 1.000	*k* = −8→8
8084 measured reflections	*l* = −20→20

### Refinement

**Table:**

Refinement on *F*^2^	Primary atom site location: structure-invariant direct methods
Least-squares matrix: full	Secondary atom site location: difference Fourier map
*R*[*F*^2^ > 2σ(*F*^2^)] = 0.068	Hydrogen site location: inferred from neighbouring sites
*wR*(*F*^2^) = 0.163	H atoms treated by a mixture of independent and constrained refinement
*S* = 1.06	*w* = 1/[σ^2^(*F*_o_^2^) + (0.0542*P*)^2^ + 0.8793*P*] where *P* = (*F*_o_^2^ + 2*F*_c_^2^)/3
1849 reflections	(Δ/σ)_max_ < 0.001
122 parameters	Δρ_max_ = 0.37 e Å^−^^3^
3 restraints	Δρ_min_ = −0.36 e Å^−^^3^

### Special details

**Table:**

Geometry. All e.s.d.'s (except the e.s.d. in the dihedral angle between two l.s. planes) are estimated using the full covariance matrix. The cell e.s.d.'s are taken into account individually in the estimation of e.s.d.'s in distances, angles and torsion angles; correlations between e.s.d.'s in cell parameters are only used when they are defined by crystal symmetry. An approximate (isotropic) treatment of cell e.s.d.'s is used for estimating e.s.d.'s involving l.s. planes.
Refinement. Refinement of *F*^2^ against ALL reflections. The weighted *R*-factor *wR* and goodness of fit *S* are based on *F*^2^, conventional *R*-factors *R* are based on *F*, with *F* set to zero for negative *F*^2^. The threshold expression of *F*^2^ > σ(*F*^2^) is used only for calculating *R*-factors(gt) *etc*. and is not relevant to the choice of reflections for refinement. *R*-factors based on *F*^2^ are statistically about twice as large as those based on *F*, and *R*- factors based on ALL data will be even larger.

### Fractional atomic coordinates and isotropic or equivalent isotropic displacement parameters (Å^2^)

**Table:**

	*x*	*y*	*z*	*U*_iso_*/*U*_eq_	
P1	0.95245 (13)	0.08087 (17)	0.17417 (6)	0.0411 (3)	
F2	0.8739 (4)	0.0534 (5)	0.25799 (19)	0.0871 (10)	
F5	1.0375 (4)	0.1087 (5)	0.09153 (17)	0.0832 (10)	
F3	0.7745 (3)	0.1086 (5)	0.1178 (2)	0.0921 (11)	
F4	0.9416 (4)	−0.1629 (4)	0.1595 (2)	0.0809 (9)	
F6	1.1318 (4)	0.0484 (6)	0.22919 (18)	0.0884 (11)	
F1	0.9643 (5)	0.3248 (5)	0.1878 (2)	0.1043 (13)	
N1	0.1232 (4)	0.0309 (5)	0.4452 (2)	0.0373 (8)	
C3	0.1606 (5)	−0.0920 (6)	0.5267 (2)	0.0430 (10)	
H3A	0.2412	−0.2008	0.5206	0.052*	
H3B	0.2089	0.0001	0.5733	0.052*	
C2	−0.0059 (5)	0.1900 (6)	0.4520 (2)	0.0418 (10)	
H2A	−0.0324	0.2650	0.3979	0.050*	
H2B	0.0370	0.2908	0.4963	0.050*	
C1	0.2776 (5)	0.1300 (7)	0.4226 (3)	0.0585 (13)	
H1A	0.2493	0.2095	0.3704	0.088*	
H1B	0.3561	0.0223	0.4144	0.088*	
H1C	0.3262	0.2215	0.4683	0.088*	
O1	0.0661 (4)	0.6583 (5)	0.34880 (19)	0.0503 (8)	
H1	0.144 (4)	0.639 (9)	0.320 (3)	0.09 (2)*	
H2	−0.027 (3)	0.648 (9)	0.317 (3)	0.11 (2)*	
H3	0.092 (5)	−0.064 (7)	0.406 (3)	0.050 (12)*	

### Atomic displacement parameters (Å^2^)

**Table:**

	*U*^11^	*U*^22^	*U*^33^	*U*^12^	*U*^13^	*U*^23^
P1	0.0418 (6)	0.0436 (6)	0.0374 (6)	0.0029 (5)	0.0049 (4)	0.0021 (5)
F2	0.106 (3)	0.100 (2)	0.0671 (19)	0.0070 (19)	0.0484 (18)	0.0093 (17)
F5	0.081 (2)	0.123 (3)	0.0512 (17)	0.0102 (18)	0.0271 (15)	0.0156 (16)
F3	0.0463 (17)	0.120 (3)	0.102 (2)	0.0144 (17)	−0.0091 (16)	0.036 (2)
F4	0.091 (2)	0.0488 (17)	0.100 (2)	0.0019 (15)	0.0079 (18)	−0.0138 (15)
F6	0.0546 (18)	0.129 (3)	0.0720 (19)	−0.0122 (17)	−0.0164 (15)	0.0205 (19)
F1	0.141 (3)	0.0461 (19)	0.135 (3)	−0.0075 (18)	0.052 (3)	−0.0085 (18)
N1	0.0398 (19)	0.0371 (19)	0.0347 (18)	−0.0072 (14)	0.0053 (14)	−0.0064 (15)
C3	0.042 (2)	0.043 (2)	0.042 (2)	0.0057 (18)	−0.0009 (17)	0.0016 (18)
C2	0.055 (3)	0.034 (2)	0.036 (2)	0.0004 (18)	0.0031 (18)	0.0050 (17)
C1	0.054 (3)	0.065 (3)	0.060 (3)	−0.023 (2)	0.021 (2)	−0.009 (2)
O1	0.052 (2)	0.0492 (19)	0.0513 (18)	−0.0009 (15)	0.0137 (17)	−0.0023 (14)

### Geometric parameters (Å, º)

**Table:**

P1—F2	1.569 (3)	C3—H3A	0.9700
P1—F4	1.570 (3)	C3—H3B	0.9700
P1—F1	1.569 (3)	C2—C3^i^	1.492 (6)
P1—F3	1.578 (3)	C2—H2A	0.9700
P1—F5	1.583 (3)	C2—H2B	0.9700
P1—F6	1.583 (3)	C1—H1A	0.9600
N1—C2	1.476 (5)	C1—H1B	0.9600
N1—C3	1.491 (5)	C1—H1C	0.9600
N1—C1	1.501 (5)	O1—H1	0.845 (10)
N1—H3	0.88 (4)	O1—H2	0.844 (10)
C3—C2^i^	1.492 (6)		
			
F2—P1—F4	89.64 (18)	C1—N1—H3	106 (3)
F2—P1—F1	91.11 (18)	C2^i^—C3—N1	110.7 (3)
F4—P1—F1	179.25 (19)	C2^i^—C3—H3A	109.5
F2—P1—F3	91.30 (19)	N1—C3—H3A	109.5
F4—P1—F3	90.21 (18)	C2^i^—C3—H3B	109.5
F1—P1—F3	89.8 (2)	N1—C3—H3B	109.5
F2—P1—F5	178.11 (18)	H3A—C3—H3B	108.1
F4—P1—F5	90.61 (18)	N1—C2—C3^i^	111.5 (3)
F1—P1—F5	88.65 (19)	N1—C2—H2A	109.3
F3—P1—F5	90.57 (17)	C3^i^—C2—H2A	109.3
F2—P1—F6	89.52 (18)	N1—C2—H2B	109.3
F4—P1—F6	88.61 (18)	C3^i^—C2—H2B	109.3
F1—P1—F6	91.4 (2)	H2A—C2—H2B	108.0
F3—P1—F6	178.6 (2)	N1—C1—H1A	109.5
F5—P1—F6	88.61 (17)	N1—C1—H1B	109.5
C2—N1—C3	110.1 (3)	H1A—C1—H1B	109.5
C2—N1—C1	111.3 (3)	N1—C1—H1C	109.5
C3—N1—C1	111.5 (3)	H1A—C1—H1C	109.5
C2—N1—H3	113 (3)	H1B—C1—H1C	109.5
C3—N1—H3	104 (3)	H1—O1—H2	110 (2)

Symmetry code: (i) −*x*, −*y*, −*z*+1.

### Hydrogen-bond geometry (Å, º)

**Table:**

*D*—H···*A*	*D*—H	H···*A*	*D*···*A*	*D*—H···*A*
O1—H1···F6^ii^	0.85 (1)	2.18 (2)	3.008 (5)	167 (5)
O1—H2···F3^iii^	0.84 (1)	2.42 (4)	2.923 (5)	119 (4)
N1—H3···O1^iv^	0.88 (4)	1.98 (4)	2.814 (4)	159 (4)

Symmetry codes: (ii) −*x*+3/2, *y*+1/2, −*z*+1/2; (iii) −*x*+1/2, *y*+1/2, −*z*+1/2; (iv) *x*, *y*−1, *z*.
